# The prevalence and characteristics of misophonia in Ankara, Turkey: population-based study

**DOI:** 10.1192/bjo.2021.978

**Published:** 2021-08-06

**Authors:** Cengiz Kılıç, Gökhan Öz, Kezban Burcu Avanoğlu, Songül Aksoy

**Affiliations:** Department of Psychiatry, Hacettepe University, Turkey; and Stress Assessment and Research Center, Hacettepe University, Turkey; Stress Assessment and Research Center, Hacettepe University, Turkey; Stress Assessment and Research Center, Hacettepe University, Turkey; and Department of Psychiatry, Hacettepe University, Turkey; Department of Audiology, Hacettepe University, Turkey

**Keywords:** Misophonia, decreased sound tolerance, tinnitus, epidemiology, hyperacusis

## Abstract

**Background:**

Misophonia is defined as significant distress (anger, distress or disgust) when exposed to certain sounds that would not affect most people, such as lip smacking or gum chewing. Although misophonia is common, the aetiology, prevalence and effective treatments are largely unknown.

**Aims:**

Based on our proposed diagnostic criteria, we examined the prevalence of misophonia and its relationship with clinical and demographic variables in a large representative population sample.

**Method:**

We used a household sample (*N* = 541) of all residents aged >15 years, living in 300 homes randomly selected in Ankara city centre, Turkey. All participants were assessed at their homes by trained interviewers, for sociodemographic variables, misophonic sounds and related factors, using a semi-structured interview (the Misophonia Interview Schedule) developed for the current research.

**Results:**

The current misophonia diagnosis prevalence was 12.8% (*n* = 69 of 541), although 427 (78.9%) participants reported at least one sound that was distressing. The mean number of misophonic sounds was 8.6 (s.d. 8.9, range 0–44); the figure was 17.6 in those with misophonia compared with 7.3 in those without misophonia. Of those with misophonia, only 5.8% contacted services for their condition. Predictors of misophonia diagnosis included younger age, family history of misophonia and previous contact with mental health services.

**Conclusions:**

Our study showed that misophonia is common in the general population, may cause significant disruption in daily life and is undertreated. Although more evidence is needed to classify misophonia as a psychiatric disorder, our findings support others who claim that the condition belongs to the group of mental disorders.

Misophonia is characterised by excessive discomfort and annoyance in response to sounds that do not annoy most people, as well as significant levels of anger, disgust or distress upon exposure to such sounds.^1–3^ Misophonic sounds (triggers) are most commonly those made by other people, such as lip smacking, eating, chewing, snoring or breathing. Sounds not originating directly from other people, such as a fork scratching a plate, a ticking clock or dripping water, can also be misophonic. Although dislike of certain sounds is common in the general population, misophonia is a distinct clinical syndrome with serious consequences; those with misophonia often experience significant social impairment at home, work, school and in social settings.^4,5^ Misophonia has been considered as a decreased sound tolerance syndrome, alongside hyperacusis and phonophobia.^2^

Although misophonia is currently not classified as a mental disorder in the ICD-11, interest in misophonia among psychiatrists and psychologists is increasing. For example, the Web of Science includes only 36 articles on misophonia that were published between 1996 and 2015, compared with 79 articles published between 2016 and 2020. Further, most recent publications on misophonia were published in psychiatry and psychology journals, not in audiology journals, as was previously the case. Based on the intense feelings of anger and anxiety upon exposure to misophonic sounds, avoidance coping strategies similar to those seen in other mental disorders, high rates of psychiatric comorbidity and an intact auditory system in those with misophonia, some researchers have posited that misophonia should be classified as a mental disorder.^1,3,6^

Little is known about the prevalence of misophonia in the general population. Existing prevalence studies included only college students or clinical populations, which is probably the main reason for differing prevalence estimates (ranging from 6 to 20%).^7–11^ The paucity of research on misophonia in the general population prevents making a definitive conclusion about its prevalence.^5,12^ Moreover, there is lack of consensus concerning misophonia diagnostic criteria, although two diagnostic criteria have been proposed. The first proposed criteria set,^1,13^ for example, suggests that misophonic sounds must be of human origin (more specifically, an oral or nasal sound), whereas some common misophonic sounds originate from animals and machines (a clock ticking, an insect buzzing and a fork scratching a plate). The second proposed criteria set, as well as the first, asserts that misophonic sounds should lead to an impulsive, aversive physical reaction.^3^ That said, most patients with misophonia in our clinical practice do not report any physical response to misophonic sounds. Both sets of criteria include angry outbursts or loss of control upon exposure to misophonic sounds, which are rare phenomena; most patients with misophonia do not report loss of control and can control the manifestation of such outbursts.^6,14^ Finally, both research groups^1,3^ included insight in their criteria, suggesting that patients with misophonia view their symptoms as unreasonable, excessive or inappropriate (i.e. they have good insight and know their reactions and excessive emotions are not normal, but are symptoms of an illness). This is, however, not what we usually observe in our clinical practice; most patients with misophonia claim that people who produce misophonic sounds are disrespectful and that their own reactions are appropriate.

## Study aim

The accumulated knowledge on misophonia remains very limited. There is a lack of consensus regarding what the typical clinical presentations are and what the true prevalence is. Large-scale epidemiological studies in representative general population samples can address some of these problems.^5^ On the other hand, any study attempting to provide a prevalence figure will need to utilise diagnostic criteria, or develop such criteria, as there are no widely accepted misophonia diagnostic criteria. To address some of these shortcomings, the present study aimed to determine the prevalence of misophonia in a large and representative general population sample, using a detailed assessment instrument that can facilitate clinical diagnosis. Well-defined criteria to diagnose misophonia were proposed, following the suggestions made by experts.^6^ The present exploratory study, therefore, was not based on any specific hypotheses. In accordance with the proposed criteria, the study aimed to determine an estimate of the prevalence of misophonia, as well as determine the correlates of the proposed misophonia diagnostic criteria.

## Method

### Sample

This cross-sectional study included a random sample representative of the general population of the centre of Ankara, Turkey. Data were collected in 2015. Ankara is the capital of Turkey and had a population of 4 million at the time of data collection. As a registry of all households was not available at the time of the study, a sampling consultant suggested using bus stops as the sampling frame. Public buses in the city centre are operated by the municipality and are evenly distributed across the city in proportion to population density. In total, 60 starting points (bus stops) were randomly selected, for which five households each were visited (for a total of 300 households). The target sample, therefore, was all people aged >15 years living in the 300 households. Among the 300 households contacted, 41 (13.6%) refused to participate in the study. Among the 710 residents aged >15 years living in the remaining 259 households, 528 (74.4%) agreed to participate in the study and 17 (2.4%) were excluded because of intellectual disability or dementia. In addition, 70 people known to live at the selected households could not be contacted in person despite making up to three visits to the house, but 15 of them agreed to be participate via telephone (they were not available for face-to-face interviewing).

**Fig. 1:**
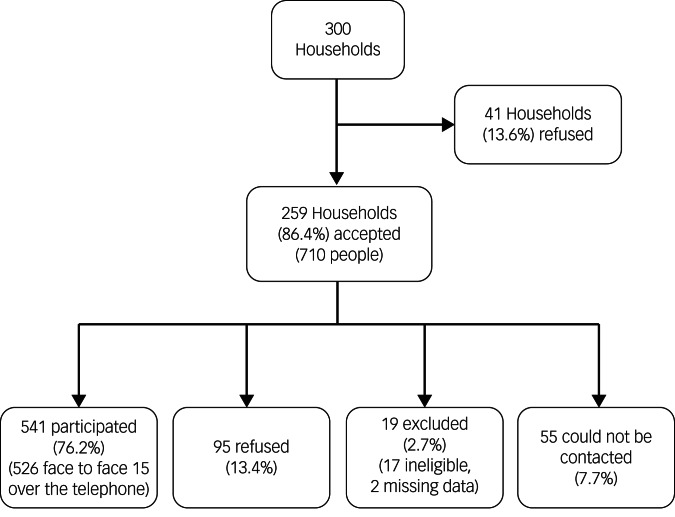
Recruitment of participants.

All consenting household members aged >15 years were considered eligible. Exclusion criteria included any conditions associated with communication problems (intellectual disability, speech or hearing problems, dementia). Ineligibility was determined by asking each household contact if anyone in the household had communication problems. Some respondents were excluded by the interviewer based on their behaviour. One of the data collection team members was a psychiatry registrar that also helped determine if a household member should be coded as eligible or excluded. In total, 86.4% of the target households were contacted and interviews were completed with 76.1% of the contacted households. In all, two interviews were excluded because of incomplete data. The final sample included 541 individuals (Fig. 1).

### Assessments

During each interview, demographic data were collected first, including age, gender, marital status and level of education. Next, the Misophonia Interview Schedule^15^ (MIS), a semi-structured interview developed for the current study, was administered. The MIS includes the Misophonia Checklist^15^ (MCL; a list of 50 misophonic sounds), as well as an additional five items for assessing the presence of misophonia diagnosis and 15 items assessing other, misophonia-associated symptoms (see supplement). The MCL assesses the level of distress when exposed to misophonic sounds during the previous month, using a four-point Likert-type scale (0, none; 1, low; 2, moderate; 3, high). The MCL was developed for this study by the researchers, and was based on a review of the relevant literature, symptom profiles of patients with misophonia treated at our psychiatry and audiology out-patient clinics, and discussions with clinicians with experience assessing patients with misophonia.

The respondents were asked to select the two misophonic sounds they found most distressing, and then complete the remainder of the MIS items designed to assess symptoms required for a diagnosis of misophonia. The interviewers were instructed to carefully confirm that reported misophonic sounds were, in fact, misophonic in nature. For example, mosquito buzzing was not coded as misophonic if the associated distress was solely related to fear of being stung.

Using the MCL, the Misophonic Sound Count (MSC) was created, which is the sum of the 51 MCL items (50 plus ‘any others?’ item) after recoding the responses into binary form (none and low indicating absent; moderate and high indicating present). The reliability indices of the MCL are excellent (Cronbach's alpha of 0.95 and Gutmann's split-half of 0.89). Respondents that reported one or more MCL sounds (scored as moderate or high) were asked additional questions concerning the onset, frequency and duration of the sounds; the types of emotional reactions they experienced in response to those sounds; and any disability associated with their misophonia symptoms. For those who did not report any MCL sound (scored as moderate or high), the MIS was ended (see the supplement for details of the diagnostic process).

The last part of the interview was used to collect data on the required symptoms for a diagnosis of misophonia (i.e. emotional reaction to misophonic sounds, strategies for coping with misophonic sounds and interference with activities caused by misophonic sounds). Other variables that were assessed included misophonia-related phenomena, such as a history of tinnitus, the presence of non-auditory triggers, a history of contact with mental health services, current use of psychotropics and a history of common mental disorders (i.e. depression, panic, attention-deficit hyperactivity disorder and phobias). Past psychiatric diagnoses were coded by response to the question: ‘Were you ever given a diagnosis of …. by a psychiatrist?’

### Procedure

We made a list of all available households before data collection started. The listing procedure began at each selected bus stop, and moving in a predefined direction (walking a spiral route heading north), the first five available households were selected. Consequently, 300 randomly selected households were visited by trained interviewers. Interviews were conducted at the respondents’ homes by nine trained interviewers, including three psychiatry registrars. The nine interviewers formed groups of three or four that visited the selected households. The interview groups were gender-balanced and, whenever possible, included at least one psychiatry registrar.

All interviews were conducted face to face, except for 15 that were conducted via the telephone. All interviews were conducted during a single session of about 40 min. It was a pencil-and-paper assessment. At the start of each interview session (both in person and via telephone), text was read aloud to the eligible respondent. The interviewer checked the box ‘agree’ if the respondent agreed to participate and checked the box ‘not agree’ if the respondent declined to participate, noting reasons for not participating if any were provided. The study protocol was approved by the Hacettepe University Ethics Committee (approval number: GO 14/552-24).

### Proposed diagnostic criteria

Based on our clinical observations of patients with misophonia, a comprehensive review of the misophonia literature and discussions with colleagues, diagnostic criteria for misophonia were proposed following the line of reasoning used for diagnosing common mental disorders.^6,16^ Below are the proposed criteria and justification for including each criterion.

- Presence of one or more misophonic sounds (trigger) causing significant distress upon exposure: to meet this criterion, one or more of the 51 MCL items should be scored as moderate or high.
- Significant emotional reaction upon misophonic sound exposure: although patients with misophonia report a wide array of disturbing emotions upon exposure to misophonic sounds, only anger, disgust and distress were considered. As most of our clinical patients with misophonia described these negative feelings as ‘intense’, we required an MCL score of high for any of these three emotions.
- Significant degree of coping strategies: almost all of our clinical patients with misophonia describe active (warning or quarrelling with the source person) or passive (avoiding misophonic sounds or using ear plugs) coping strategies, which are an indication of the severity of symptoms. We required one or more coping strategy (out of a list of eight) to be present to a significant degree (i.e. scored as ‘often’ or ‘always’).
- Symptoms should be severe enough to cause significant interference with daily activities: to meet this criterion, the presence of one or more of the following interference items must be answered as yes: ‘Do you avoid certain places because of symptoms?’; ‘Are there things you can't do because of symptoms?’; ‘Do symptoms disrupt your relations with others?’.

### Statistical analysis

Data were analysed with SPSS Statistics for Windows, version 25 (IBM Corporation, New York, USA). The *χ*^2^-test was used to determine and compare prevalences of the proposed misophonia diagnostic criteria between groups, using the following variables: gender, education, marital status, employment status, history of tinnitus, family history of misophonia, use of services and use of psychotropics. Mean age and mean number of misophonic sounds were compared between diagnostic groups, using *t*-tests.

Correlates of the diagnosis of misophonia were examined with binomial logistic regression. The selection of variables included in the explanatory variable set was based on clinical experience with patients with misophonia and a review of the literature. In addition to standard demographic variables (i.e. gender, age, level of education and marital status), a history of tinnitus, family history of misophonia and a history of contact with mental health services were analysed. The level of statistical significance was set at *P* < 0.05.

## Results

The study included 541 participants, of which 314 (58%) were female and 227 (42%) were male. Mean age of the participants was 43.5 (s.d. 18.2, range 15–88) years and 42 (7.8%) were aged <18 years. The description of the study sample according to misophonia status is presented in Table 1. Mean number of misophonic sounds reported for each participant was 8.6 (s.d.8.9, range 0–44). In total, 114 (21.1%) participants did not report any misophonic sounds (all MCL items were scored as none or low). In all, 427 (78.9%) participants scored one or more MCL item as moderate or high. The mean number of misophonic sounds (MSC score) reported by those with misophonia was 17.6 (s.d. 9.1, range 1–40), compared with 7.3 (s.d. 8.1, range 0–44) among those without misophonia. The MSC score was negatively correlated with age (*r* = –0.25, *P* = 0.001).

**Table 1:** Description of the study sample by misophonia status

	Misophonia only (*n* = 69)	No misophonia (*n* = 472)	Total (*N* = 541)
Gender, female**	51 (73.9)	263 (55.7)	314 (58.0)
Age groups, years**			
15–24	21 (30.4)	85 (18.0)	106 (19.6)
25–44	26 (37.7)	137 (29.0)	163 (30.1)
45–64	20 (29.0)	175 (37.1)	195 (36.0)
≥65	2 (2.9)	75 (15.9)	77 (14.2)
Education, college graduate	17 (24.6)	135 (28.6)	152 (28.1)
Marital status, married*	37 (53.6)	312 (66.1)	349 (64.5)
Employment status, employed	18 (26.1)	166 (35.2)	184 (34.0)
History of tinnitus	30 (43.5)	177 (37.6)	207 (38.3)
Family history of misophonia**	30 (43.5)	67 (14.3)	97 (18.1)
Past contact with mental health services**	33 (47.8)	129 (27.4)	162 (30.1)
Current use of psychotropics**	16 (23.2)	52 (11.1)	68 (12.6)
Total	69 (12.8)	472 (87.2)	541 (100.0)
Mean (s.d.) age**	35.5 (15.6)	44.7 (18.2)	43.5 (18.2)
Mean (s.d.) number of misophonic sounds**	17.6 (9.1)	7.3 (8.1)	8.6 (8.9)

All data are displayed as *n* (%), unless otherwise indicated.

** *P* < 0.01.

### Correlates of misophonia

Based on the proposed diagnostic criteria, the prevalence of misophonia in the study population (*N* = 541) was 12.8% (*n* = 69). Female gender, younger age and not being married seemed to be related to higher rates of misophonia. History of tinnitus did not relate to misophonia diagnosis. Almost half (43.5%) of those with misophonia diagnosis reported that they had a first-degree relative with similar symptoms. In all, 63.8% of those with misophonia reported that their symptoms began during childhood or adolescence. In addition to reporting auditory triggers, the majority (56.5%) of those with misophonia reported that they experienced distress when they saw people eating or chewing from a distance, despite not being able to hear any sound (visual trigger). Similarly, 42.0% of those with misophonia were distressed by visual triggers, such as people shaking feet.

Those with misophonia were more likely than those without misophonia to have had a (self-reported, psychiatrist-diagnosed) history of attention-deficit hyperactivity disorder (20.3 *v.* 7.3%, *P* = 0.001), obsessive–compulsive disorder (15.9 *v*. 5.0%, *P* = 0.001), bipolar disorder (7.2 *v*. 1.4%, *P* = 0.003), substance use disorder (4.3 *v*. 0.6%, *P* = 0.007), conversion disorder (13.0 *v*. 4.2%, *P* = 0.004) and attempted suicide (5.8 *v*. 1.7%, *P* = 0.04). Those with misophonia were also more likely to be currently using psychotropic medication (23.2 *v*. 11.2%, *P* = 0.007). A history of contact with mental health services for any psychological problem was more common among those with misophonia than those without (47.8 *v*. 28.7%, *P* = 0.002), although only four (5.8%) of the 69 participants with misophonia had contact with mental health services because of symptoms of misophonia.

Participants with misophonia generally rated their physical health to be better than their mental health: 14.5% rated their current mental health as ‘bad’ (the choices were very good, good, fair and bad), compared with 5.5% of those without misophonia. Conversely, those who rated their current physical health as ‘bad’ were lower in participants with misophonia versus those without misophonia (2.9 *v*. 5.5%). In total, 43.5% of those with misophonia reported having a first-degree-relative (parent, sibling or offspring) with misophonia, compared with 14.3% of those without misophonia. Low-level insight was common, as 65.4% of those with misophonia did not consider their condition as an illness. On the other hand, more of those with misophonia considered it a psychological versus physical problem (21.7 *v.* 2.9%, *P* = 0.01).

### Most common misophonic sounds

Table 2 shows the most commonly reported misophonic sounds by diagnostic status. Interestingly, the more commonly reported misophonic symptoms are also commonly reported by those without misophonia, such as snoring, mosquito buzzing, etc. On the other hand, some sounds less commonly reported by those with misophonia are practically not distressing at all to most people without misophonia (e.g. infant crying, sucking teeth or um-err speech). This finding may be an indication of low discriminatory power of most frequently reported misophonic sounds.

**Table 2:** Most commonly endorsed misophonic sounds by misophonia status (*N* = 541)

Misophonic sounds (causing moderate to high distress)	Misophonia (*n* = 69)	No misophonia (*n* = 472)	Total (*N* = 541)
Fly/mosquito buzzing**	51 (73.9)	181 (38.3)	232 (42.9)
Snoring**	50 (72.5)	205 (43.4)	255 (47.1)
Eating, chewing, lip-smacking sounds**	49 (71.0)	184 (39.0)	233 (43.1)
Nose sniffing**	47 (68.1)	143 (30.3)	191 (35.1)
Throat clearing**	45 (65.2)	148 (31.4)	193 (35.7)
Slurping when drinking tea, coffee, soup**	42 (60.9)	127 (26.9)	169 (31.2)
Sucking teeth**	40 (58.0)	113 (23.9)	153 (28.3)
Sound of dripping water**	39 (56.5)	139 (29.4)	178 (32.9)
Fork scratch on plate**	39 (56.5)	117 (24.8)	156 (28.8)
Gum chewing**	38 (55.1)	133 (28.2)	171 (31.6)
Infant crying**	36 (52.2)	63 (13.3)	99 (18.3)
Squeaking of door/floor/fabric**	35 (50.7)	99 (21.0)	134 (24.8)
Music or television through walls**	31 (44.9)	81 (17.2)	112 (20.7)
‘Um, uh, er’ speech**	30 (43.5)	72 (15.3)	102 (18.9)

All data are displayed as *n* (%), unless otherwise indicated.

** *P* < 0.01.

### Regression analysis

Binomial logistic regression analysis was performed with the diagnosis of misophonia as a categorical dependent variable (Table 3). Explanatory variables were chosen on the basis of relevant literature and clinical judgement: female gender (1, male; 2, female), age (15–88 years), education (0, illiterate; 3, university), marital status (0, not married; 1, married), presence of tinnitus (0, absent; 1, present), family history of misophonia (0, absent; 1, present) and previous contact with mental health services (0, absent; 1, present). The results showed that there were three significant associated factors: younger age, a history of contact with mental health services and family history of misophonia (the presence of misophonic symptoms in first-degree relatives). Misophonia was not associated with other demographic factors (gender, level of education or marital status).

**Table 3:** Predictors of misophonia status (*N* = 541, logistic regression)

	Wald *χ*^2^	*P*-value	Exp(B)	95% CI for Exp(B)	
				Lower	Upper
Gender, female	3.211	0.073	1.764	0.948	3.280
Age**	10.387	0.001	0.967	0.947	0.987
Education	1.157	0.282	0.876	0.688	1.115
Marital status, married	0.016	0.899	1.043	0.544	1.999
History of tinnitus	1.474	0.225	1.421	0.806	2.506
Family history of misophonia**	18.132	0.001	3.475	1.959	6.165
Past contact with mental health services**	8.711	0.003	2.312	1.325	4.033

Explanatory variables: female gender (1, male; 2, female), age (15–88 years), education (0, illiterate; 3, university), marital status (0, not married; 1, married), presence of tinnitus (0, absent; 1, present), family history of misophonia (0, absent; 1, present), contact with mental health services (0, absent; 1, present).

** *P* < 0.01.

## Discussion

The present study confirms earlier findings on misophonia: it is common in the general population, leads to considerable distress and interferes with daily functioning. As most cases start very early in life, it can have significant and deleterious effects during the period when young individuals are building social bonds and learning life skills. Misophonia shares several defining characteristics with other common psychiatric disorders, especially those on the phobic and obsessive–compulsive spectrums. Misophonia has much in common with other common psychiatric disorders: misophonic reactions are largely subjective (i.e. independent of the objective qualities of misophonic sounds), excessive and disproportional to the trigger, and lead to excessive coping attempts (mostly in the form of avoidance); affected individuals have a much higher rate of a history of psychiatric illness; and affected individuals have a high rate of contact with mental health services and current use of psychotropics. Moreover, in the present study, participants with misophonia described their mental health as much worse than their physical health; in fact, 21.7% of those with misophonia defined their problem as a psychiatric disorder, and very few regarded their condition as a physical disorder. It is not yet certain if misophonia should be classified as a psychiatric disorder, but evidence supporting this is accumulating.

### The prevalence of misophonia and associated factors

To the best of our knowledge, the present study is the first to determine the prevalence of misophonia in a large, representative community sample. The present study also used face-to-face interviews for data collection – a rare practice in misophonia research. The proposed diagnostic criteria diagnosed one in eight individuals (12.8%) in the general population as having misophonia. The literature includes only a few studies on the prevalence of misophonia, although none were conducted with a general population or randomised sample; most are based on self-report and online surveys of college students, or are based on convenience samples designed to elicit more individuals with misophonia. Interestingly, the only other study that reported the prevalence of misophonia based on face-to-face interviews^10^ noted a prevalence of 12.8%. Although that figure is the same as that in the present study, it must be noted that this earlier study included in-patients with depression, a population in which misophonia is expected to be more common than in the general population.

Although univariate analyses suggest that several demographic variables (female gender, being single and being younger) are related to misophonia, the only demographic variable that was a predictor of misophonia was younger age. In categorical age groups, prevalence of misophonia continually drops as age progresses. Although the prediction is a weak one, this finding is in line with the course of most mental disorders, which start early in life and subside in later years.

The present findings show that misophonia may run in families, as 43.5% of those diagnosed as misophonia reported having first-degree relatives with similar symptoms. Other studies that assessed family history of misophonia reported rates of 22−33%,^13,17^ which is lower than that observed in the present study and might be because those earlier studies were based on non-representative convenience samples. One of those studies^17^ highlights a unique aspect of family history of misophonia: participants with misophonia had more female than male relatives with the same condition. Although this sounds interesting, it is also possible that this is simply a reflection of a higher prevalence of misophonia in females in the general population. Little is known about the genetics of misophonia,^18^ and genetic epidemiology studies could help delineate the corresponding effects of nature versus nurture.

The misophonia literature suggests that childhood onset is the norm.^1,4,13,17^ In line with the literature, in most (63.8%) participants with misophonia in the present study, the symptoms of misophonia began during childhood or adolescence; nonetheless, misophonia did not correlate with the onset variable in the present sample. Although several studies have reported on the onset of misophonic symptoms, none have examined the correlates of misophonia onset, which is clearly a neglected area of research.

### Use of mental health services

Only 5.8% of the participants with misophonia in the present study had a history of contact with mental health services for misophonic symptoms. Unfortunately, there is no other comparable study in the literature, but it is intuitive to think that the rate for such contact in the general population is low. The low rate of help-seeking for misophonia in the present study might have been because of a lack of awareness about the condition or the absence of an established and effective treatment, both of which can have a negative effect on help-seeking behaviour.^13^ There are some reports of the successful treatment of misophonia, although overall, the evidence is not convincing.^19,20^ Anecdotal reports show that some patients benefit from cognitive–behavioural therapy.^21^ Another reason for the low rate of contact with mental health services among those with misophonia may be the high rate of low-level insight: those with misophonia that do not think that anything is wrong may not be motivated to seek help. Finally, as is the case with many phobias, avoidance may mask symptoms, thereby decreasing the need to have contact with mental health services.^22^

### Limitations

The present study has several limitations. Although face-to-face interviews were conducted, they did not include a full clinical psychiatric interview. Using a clinical interview would have facilitated assessment of other psychiatric disorders, and psychiatric comorbidity is known to be high among patients with misophonia.^1,13,23^ Use of a clinical interview would also have enabled the addition of another criterion to the proposed diagnostic criteria: ‘symptoms not better explained by another disorder’.

Interviewers should have asked the participants if they experienced distress every time they were exposed to misophonic sounds, and those that did not should not have been diagnosed as misophonia. Although the literature and our clinical experience suggest misophonia has a chronic course, the duration of symptoms was not assessed. The literature on the duration of the symptoms in misophonia is lacking. It is therefore reasonable to add a diagnostic criterion requiring that the symptoms must persist for more than 6 months, to exclude those with transitory symptoms (i.e. resulting from an ear infection or flu). Finally, the study assessed the symptoms of misophonia during the previous month only. Although the MIS included an item regarding the onset of symptoms, the course of misophonia was not assessed in detail.

### Recommendations

First, we suggest using interviews for assessing misophonia as opposed to self-report questionnaires. Many sounds that might be rated on a self-report questionnaire as misophonic may not actually be misophonic (i.e. a vacuum cleaner and snoring) or may be anxiety-provoking cues (i.e. dog barking for a person with a dog phobia or the sound of breathing for a rape victim) and, therefore, should not contribute to a diagnosis of misophonia. Our experience showed that a trained interviewer can successfully avoid such false positives. A psychiatric interview will have the additional benefit of providing reliable rates of comorbid diagnoses.

Second, some misophonic sounds (such as snoring or mosquito buzzing), although commonly endorsed by those with misophonia, may not be very useful in discriminating those with misophonia from those without misophonia. Future studies may help us select among the long list of misophonic sounds, to construct a limited list of items with maximum discriminatory power.

Third, subtypes of misophonia deserve the attention of future researchers. We still know very little about this condition, and it is possible that there are different types of misophonic syndromes that are more common in women than men, or in some countries than others.

Finally, the diagnostic criteria we are proposing are preliminary and must be verified by additional research. Follow-up studies are required to show the invariance of the proposed diagnostic criteria in patients with misophonia.

## Data Availability

The data that support the findings of this study are available upon reasonable request from the corresponding author, C.K., with the permission of Hacettepe University.

## Supplementary material

For supplementary material accompanying this paper visit http://dx.doi.org/10.1192/bjo.2021.978. click here to view supplementary material
