# Supplementary material for: The prevalence and characteristics of misophonia in Ankara, Turkey: population-based study
Source: BJPsych Open. 2021 Aug 6;7(5):e144. doi: 10.1192/bjo.2021.978 (PMC8358974; doi:10.1192/bjo.2021.978)
Supplement: Supplementary file 1 [file S2056472421009789sup001.docx]

**Supplement**

**Misophonia Interview Schedule (MIS)**

**(Öz & Kılıç 2014)**

**The Measure**

Below is a description of the interview used in this study to diagnose misophonia. The Misophonia Interview Schedule (MIS) was developed by the researchers specifically for this study and was based on our clinical experience, a literature review and consultation/brainstorming sessions within the research team, which included psychiatrists and audiologists. The MIS was administered by trained individuals. Although it can be used as a questionnaire (i.e., as self-report instrument), we think using it in the interview format has advantages. The MIS includes the Misophonia Checklist (MCL)—a list of 51 potentially misophonic sounds (50 items plus “any other?” item). The MCL (see below) is used to assess the presence of reactions to those 51 sounds, as well as the degree of distress caused by each. We decided to be over-inclusive with the list of potential misophonic sounds, as little was known about misophonia when the study was designed in 2014. We therefore included typical misophonic sounds reported in the literature (i.e., throat clearing and lip-smacking), as well as less commonly reported sounds (computer mouse clicking and scratching skin). We think that future studies can eliminate some of the less commonly reported misophonic sounds.

Although the MCL was first developed as a self-report instrument, observations of misophonic patients with little insight convinced us to use it as an interviewer-administered instrument. Many sounds rated by individuals as misophonic on a self-report questionnaire may not actually be misophonic (i.e., vacuum cleaner and snoring), or may be anxiety-provoking cues (i.e., dog barking for a dog phobic or the sound of breathing for a rape victim) and, therefore, should not contribute to the diagnosis of misophonia. Interviewers were advised to be alert to misunderstandings by each participant. Relevant details appear below.

**The Interview and Diagnostic Processes**

The MIS begins with the MCL. After reading aloud each item and recording the degree of disturbance/distress caused by each, the interviewer decides if the respondent qualifies for further questioning.

| a. Presence of ≥1 misophonic sounds (triggers) causing significant distress upon exposure. To meet this criterion ≥1 of the 51 MCL items should be scored as moderate or high. |
| --- |

Those that reported one or more MCL sound caused a moderate or high degree of disturbance were administered the second part of the MIS. The number of reported misophonic sounds is not necessarily associated with the severity of misophonia or the risk of the diagnosis of misophonia. The presence of just one misophonic sound is sufficient for a diagnosis of misophonia. What we frequently see in clinical practice is that individuals that report one or two misophonic sounds can suffer greater disability than those reporting three or more misophonic sounds.

The most important point we stress when training MIS interviewers is differentiation between sounds that count towards a diagnosis of misophonia (misophonic sounds) and those that do not. Typical examples to those that are problematic include mosquito buzzing, dog barking and breathing sounds. Interviewers are instructed to be certain that a respondent is distressed both by a particular sound and the unwanted consequences associated with the sound. The sound of breathing does not qualify as a misophonic sound, for example, if the reaction to it is associated with the trauma of rape. Similarly, mosquito buzzing is not considered a misophonic sound if the distress it causes is solely related to the possibility of being stung. Finally, dog barking is not a misophonic sound for dog phobic individuals. A similar problematic example during the Covid-19 pandemic is a respondent expressing annoyance at the sound of coughing or sneezing; the interviewer must make certain that the annoyance is not related to anxiety about becoming infected.

When no disturbing MCL sounds or MCL sounds with a rating of moderate or high are reported the MIS interview stops; there is no need to ask additional questions to establish a diagnosis. For those who do report ≥1 disturbing MCL sounds scored as moderate or high, however, the interviewer asks an additional 20 diagnostic questions. After ascertaining that a respondent scored ≥1 MCL sound as moderate or high, we limit the reported number of such sounds to two by asking those that reported ≥2 misophonic sounds to select the two most disturbing sounds. We do this for three reasons: 1. A respondent that reports ≥2 misophonic sounds may find it difficult to respond to questions concerning the degree of distress caused by the sounds, as each sound elicits different degrees of distress. 2. It is easier to assess the change in response to two misophonic sounds over time than that for 10 sounds. 3. The number of misophonic sounds is limited to 2 and not 1 so as to safeguard against the possibility that an individual may choose a non-misophonic (or non-specific) sound as the most disturbing. So, for example snoring, which can be a misophonic sound, is so common a sound that it does not have a high discriminatory value. It is, therefore, more valuable to require a second sound, say gum chewing, to facilitate assessment of change over time.

| b. Significant emotional reaction upon exposure. The degree of the experience of ≥1 negative emotions upon exposure to misophonic sounds must be scored as high. |
| --- |

Next, we ascertain that the selected/reported misophonic sounds cause extreme negative emotions most commonly in the form of anger, discomfort, disgust or fear (note: it may be that such emotional responses are not experienced because of avoiding the sounds). Our clinical experience and the literature confirm that fear is rarely reported. We therefore include the three more common negative emotions in criterion b. The degree of each emotion experienced was scored as none, low, moderate and high. The negative emotions assessed are common; therefore, ≥1 negative emotion experienced must be scored as high.

| c. Significant use of coping strategies. Among the eight coping strategies assessed, ≥1 must be scored as “often” or “almost always”. |
| --- |

Next, several coping strategies commonly employed by misophonia patients were assessed. The frequency of eight common coping strategies are scored as follows: never; sometimes; often; almost always; not applicable-NA). A score of “often” or “almost always” to ≥1 of these items is required for criterion c.

| Strategies | Frequency | | | | |
| --- | --- | --- | --- | --- | --- |
| 9.1. Leaving the setting where there is a trigger sound | NA | Never | Sometimes | Often | Almost always |
| 9.2. Avoiding circumstances where there may be a trigger sound | NA | Never | Sometimes | Often | Almost always |
| 9.3. Trying to distract attention away from trigger sound | NA | Never | Sometimes | Often | Almost always |
| 9.4. Producing noise to block out the trigger sound | NA | Never | Sometimes | Often | Almost always |
| 9.5 Warning the person or silencing the trigger | NA | Never | Sometimes | Often | Almost always |
| 9.6. Quarrelling with a person producing a trigger sound | NA | Never | Sometimes | Often | Almost always |
| 9.7. Using ear-plugs | NA | Never | Sometimes | Often | Almost always |
| 9.8. Listening to music with earphones | NA | Never | Sometimes | Often | Almost always |

.

| d. Symptoms should be so severe as to cause significant interference with daily activities. To meet this criterion ≥1 of the three interference items should be rated as “yes”. |
| --- |

Finally, the level of interference with daily activities due to misophonic sounds was assessed; a rather unconventional approach was used for assessing this. Participants were asked three questions (see below) answered as yes or no and responding “yes” to just one was enough to rate interference with daily activities (criterion d) as present. What we had originally planned was to ask only one question (“do your misophonia symptoms significantly affect your daily activities?”), as is usual practice according to ICD and DSM. Based on our clinical observations, patients with severe misophonia often report little or no interference, which led to the revision of this criterion. It was concluded that reporting little or no interference was due to excessive avoidance that masks such interference. During the standardization practices prior to conducting the interviews in the field and in clinical practice we saw several misophonia patients that responded “none” or “a little” to the interference question, but answered “yes” to ≥1 of the three alternative interference questions shown below.

| Do you avoid certain places because of sounds? | NO | YES |
| --- | --- | --- |
| Are there things you cannot do because of these sounds? | NO | YES |
| Did these sounds disrupt your relations with others? | NO | YES |

The interviewers were trained to detect false negative responses when assessing this criterion and were instructed to provide examples as necessary. For example, if a participant answers “no” to all three above questions the interviewer is to provide examples based on information obtained from previous positive responses. A typical example would be “you said you dreaded the sound of a fork scratching on a plate; did this cause you to avoid going to restaurants?”

**Misophonia Checklist (MCL)**

(Öz & Kılıç 2014)

Below are sounds that some people may find annoying. Please review all and select the degree of distress caused by each during the previous month.

| Distressing sound | Degree of distress | | | |
| --- | --- | --- | --- | --- |
| 1. Eating, chewing, smacking sounds | None | Low | Moderate | High |
| 1. Chewing gum | None | Low | Moderate | High |
| 1. Cracking sunflower (or similar) seeds | None | Low | Moderate | High |
| 1. Eating a peach/pear/apple | None | Low | Moderate | High |
| 1. Eating a cucumber or carrot | None | Low | Moderate | High |
| 1. Nasal or oral breathing | None | Low | Moderate | High |
| 1. Inhalation of cigarette smoke | None | Low | Moderate | High |
| 1. Slurping when drinking tea, coffee, soup | None | Low | Moderate | High |
| 1. Whistling | None | Low | Moderate | High |
| 1. Snoring | None | Low | Moderate | High |
| 1. Hiccups | None | Low | Moderate | High |
| 1. Sneezing | None | Low | Moderate | High |
| 1. Coughing | None | Low | Moderate | High |
| 1. Throat clearing | None | Low | Moderate | High |
| 1. Nose sniffling | None | Low | Moderate | High |
| 1. Knuckle cracking | None | Low | Moderate | High |
| 1. Scratching (skin) | None | Low | Moderate | High |
| 1. Combing hair | None | Low | Moderate | High |
| 1. Nail clipping | None | Low | Moderate | High |
| 1. Dripping water | None | Low | Moderate | High |
| 1. “Um, uh, er” speech | None | Low | Moderate | High |
| 1. Your own heartbeat or pulse | None | Low | Moderate | High |
| 1. Chalk on blackboard | None | Low | Moderate | High |
| 1. Whispering | None | Low | Moderate | High |
| 1. Pencil or marker on paper | None | Low | Moderate | High |
| 1. Infant crying | None | Low | Moderate | High |
| 1. Keychain/rosary/coin rolling | None | Low | Moderate | High |
| 1. Crumpling paper/plastic bags | None | Low | Moderate | High |
| 1. Rubbing packaging foam | None | Low | Moderate | High |
| 1. Computer mouse clicks | None | Low | Moderate | High |
| 1. Keyboard clicks | None | Low | Moderate | High |
| 1. Phone notifications | None | Low | Moderate | High |
| 1. Pen clicking | None | Low | Moderate | High |
| 1. Fly/mosquito buzzing | None | Low | Moderate | High |
| 1. Fork scratching a plate | None | Low | Moderate | High |
| 1. Music leaking from someone else’s earphones | None | Low | Moderate | High |
| 1. Music or TV through walls | None | Low | Moderate | High |
| 1. Flipping pages of a book/newspaper | None | Low | Moderate | High |
| 1. Dog barking | None | Low | Moderate | High |
| 1. Squeaking of sports shoes on polished floor | None | Low | Moderate | High |
| 1. Flickering fluorescent lamp | None | Low | Moderate | High |
| 1. Boiling water | None | Low | Moderate | High |
| 1. Clock ticking | None | Low | Moderate | High |
| 1. Crumpling paper/napkin | None | Low | Moderate | High |
| 1. Sucking teeth | None | Low | Moderate | High |
| 1. Refrigerator, computer or air conditioner | None | Low | Moderate | High |
| 1. Vacuum cleaner | None | Low | Moderate | High |
| 1. Walking in the snow | None | Low | Moderate | High |
| 1. Footsteps on hard flooring | None | Low | Moderate | High |
| 1. Squeaking of door/floor/fabric | None | Low | Moderate | High |
| 1. Other ……………………………………….. | None | Low | Moderate | High |
